# 

**Published:** 1890-05

**Authors:** 


### CONTENTS.

### ORIGINAL COMMUNICATIONS—

Clinical Lecture. By Henry M. Lyman, M. D., Chicago, Ill...... 129

A Report of Two Difficult Ovariotomies. By J. J. Buckhanan. M. D., Pittsburgh........................................ 137

Report of Four Cases of Laparotomy, Two Cases of Intra-Ligamentous Cyst, One Dermoid Cyst and One Removal of Appendages, Two of the Cases Requiring Hysterectomy. By X. O. Werder, M. D., Pittsburgh.............................. 142

A Fibro-Sarcoma—The Advisability of its Removal. By W. L. Bullard, M. D., Columbus, Ga........................................ 147

### SOCIETY REPORTS—

Allegheny County Medical Society........................................ 151

Tennessee State Medical Society........................................ 157

Congress of Physicians—Forty-First Anniversary of the Georgia Medical Association........................................ 170

### CLINIC REPORTS—

Two Cases of Rare Ovarion Tumors. By Virgil O. Hardon, M. D. 179

### CORRESPONDENCE—

Our New York Letter........................................ 182

Letter from L. G. Hardman, M. D........................................ 187

### MISCELLANEOUS—

To the Survivors of the Medical Corps of the Confederate States Army........................................ 188

Invitation to the International Medico-Scientific Exhibition 190

Tenth International Medical Congress........................................ 191

